# A checklist for managed access programmes for reimbursement co‐designed by Canadian patients and caregivers

**DOI:** 10.1111/hex.12690

**Published:** 2018-04-06

**Authors:** Andrea Young, Devidas Menon, Jackie Street, Walla Al‐Hertani, Tania Stafinski

**Affiliations:** ^1^ Health Technology & Policy Unit School of Public Health University of Alberta Edmonton AB Canada; ^2^ School of Public Health The University of Adelaide Adelaide SA Australia; ^3^ Cumming School of Medicine University of Calgary Calgary AB Canada

**Keywords:** managed access programmes, orphan drugs, patient input, patient involvement, rare diseases

## Abstract

**Introduction:**

Reimbursement decisions on orphan drugs carry significant uncertainty, and as the amount increases, so does the risk of making a wrong decision, where harms outweigh benefits. Consequently, patients often face limited access to orphan drugs. Managed access programmes (MAPs) are a mechanism for managing risk while enabling access to potentially beneficial drugs. Patients and their caregivers have expressed support for these programmes and see patient input as critical to successful implementation. However, they have yet to be systematically involved in their design.

**Objective:**

The aim of this study was to co‐design with patients and caregivers a tool for the development of managed access programmes.

**Methods:**

Building upon established relationships with the Canadian Organization for Rare Disorders, the project team collaborated with patients and caregivers using the principles of participatory action research. Data were collected at two workshops and analysed using a thematic network approach.

**Results:**

Patients and caregivers co‐designed a checklist comprised of six aspects of an ideal MAP relating to accountability (programme goals); governance (MAP‐specific committee oversight, patient input, international collaboration); and evidence collection (outcome measures and continuation criteria, on‐going monitoring and registries). They recognized that health‐care resources are finite and considered disease or drug eligibility criteria for deciding when to use a MAP (eg drugs treating diseases for which there are no other legitimate alternatives).

**Conclusions:**

A patient and caregiver‐designed checklist was created, which emphasized patient involvement and transparency. Further research is needed to examine the feasibility of this checklist and roles for other stakeholders.

## 1 INTRODUCTION

Reimbursement decisions on orphan drugs (ie medicines for treating rare diseases affecting less than 5 in 10 000 people in the European Union^1^) carry significant uncertainty.^2^, ^3^ Uncertainty typically arises from a lack of high‐quality information on (i) clinical benefit, (ii) value for money, (iii) potential adoption/diffusion and (iv) affordability.^4^, ^5^ The natural histories of many rare diseases, which tend to be life‐threatening or severely debilitating, remain poorly understood, and high‐quality randomized clinical trials are often difficult to conduct because of small patient populations and limited validated outcome measures.^5^, ^6^

As uncertainty increases, so does the risk of making a “wrong decision.” Patients may be harmed and resources may be wasted when a treatment provided turns out to be ineffective or unsafe or when a treatment not provided turns out to be effective.^4^ Therefore, to manage risk while enabling access to potentially beneficial drugs, innovative ways of introducing these drugs have been developed,^7^, ^8^, ^9^ one of which may be referred to as managed access programmes (MAPs).^4^ MAPs provide patients with a drug while information needed to address uncertainties is collected to inform a definitive coverage decision. As an outcome‐based arrangement, they resemble complex patient access schemes offered through NHS England.^10^ In Canada, patients, caregivers and patient organizations have expressed support for MAPs. Further, they perceive their input to be critical to successful implementation, should such a policy option be adopted. However, they have yet to be systematically involved in their design.^4^, ^11^

## 2 OBJECTIVE

The aim of this study was to co‐design with patients and caregivers a tool for the development of managed access programmes.

## 3 METHODS

A participatory action research (PAR) approach was used. PAR requires the active involvement of researchers and participants in co‐constructing knowledge; promoting self‐ and critical awareness (which leads to individual, collective and/or social change); and building alliances for effective planning, implementation and dissemination of the research.^12^ In Canada, the Canadian Organization for Rare Disorders (CORD) represents the rare diseases community. It is comprised of more than 80 patient organizations and is recognized as the national voice of this community, advocating for appropriate access to care. In this study, we built on established relationships between CORD and members of the study team. At the same time, recent research from our group had demonstrated that there was strong interest in the CORD community in a possible role for patients and families in developing innovative approaches, such as MAPs, to improve coverage for orphan and ultra‐orphan drugs.^13^ Two workshops were held using the methods described below.

### 3.1 Study population

All patients and caregivers at two CORD Regional Forums were invited to participate in the workshops, which were part of the main Forum programme (ie no other sessions were scheduled at the same time). The Forums focused on strategies for sustainable access to therapies and explored personalized approaches to drug access. Presentations were made on assessing therapies for real‐world use, strategies for responsible use and different pathways for access, including MAPs. Prior to the Forums, participants had participated in two CORD conferences focused on improving access to therapies for rare diseases and efforts to accomplish this in other countries. They also included presentations on the challenges faced by decision‐makers in Canada and discussions around the feasibility of applying international experience to the Canadian context. CORD travel grants were provided to patients and families for the conferences and Forums, minimizing financial barriers to attendance.

### 3.2 Data collection

Workshops built upon findings from research previously undertaken in collaboration with CORD (deliberative discussions with multiple stakeholders and then patients and caregivers, followed by webinars and priority‐setting exercises with patients and families) (see Figure S1 in Appendix A for the diagram of research progression). Questions focused on the 4 main types of uncertainty that decision‐makers face (listed in the Introduction) and sought to elicit information from participants on additional sources of uncertainty and aspects of MAPs important to them (see Table S1 in Appendix A for the list of questions). Two experienced researchers facilitated both workshops, which began with a presentation on MAPs and examples of their use. Both workshops were audio‐recorded and transcribed. No training was provided prior to the workshop, but all of the participants had attended the Forum and CORD conferences.

### 3.3 Data analysis and interpretation

Transcripts were analysed using a thematic network approach,^14^ a tool for organizing the different levels of themes that emerge in a thematic analysis of qualitative data. Transcripts were first coded inductively using open coding methods.^15^ Codes were then clustered into “basic themes,” describing the premise of the coded data (eg no legitimate drug alternatives).^14^ Basic themes focusing on similar issues were further grouped into “organizing themes” (eg drug priorities for MAPs).^14^ Finally, organizing themes were grouped into “global themes,” capturing what they meant as a whole (eg best practices for an ideal managed access programme).^14^ Constant comparative analysis was used to organize codes into themes,^15^ which were subsequently mapped onto an uncertainties matrix, reflecting their link to a specific type of uncertainty. Finally, by considering how the themes could be operationalized in the implementation of a MAP, an “ideal” MAP checklist was created similar to commonly used critical appraisal tools.

The checklist was reviewed by workshop participants, minimizing opportunities for bias in the interpretation of data. It was then presented to a broader group of stakeholders who comprise members of the research and advisory teams of Promoting Rare‐Disease Innovation through Sustainable Mechanisms (PRISM; a Canadian research network through which this project was funded) for further feedback. These teams include clinicians, regulators, provincial drug plan decision‐makers and industry representatives. Based on comments received, a final version of the checklist was prepared.

## 4 RESULTS

All patients and caregivers who attended the Regional Forum participated in the workshop. They represented a range of disease types (eg cancer, non‐cancerous tumour disorders, blood disorders, metabolic disorders, connective tissue disease, endocrine disorders, lung disorders and epileptic encephalopathies) and differing levels of experience within their rare disease communities. Nine patients and three caregivers (10 females; 2 males) participated in the first workshop, and five patients and three caregivers (7 females; 1 male) participated in the second.

Through the workshops, four global themes reflecting “notions” were identified. A notion is an individual's impression of something known, experienced or imagined^16^ The notions related to patients' and caregivers' experiences living with a rare disease and accessing appropriate therapies (eg orphan drugs). Collectively these appeared to guide their views on what they considered a MAP that they felt would provide the necessary, but missing information on a new therapy. In addition to these notions, patients and caregivers also identified specific aspects of an ideal MAP. Overarching the four notions and the aspects of an ideal MAP was “sentiments,” capturing why patients valued MAPs and wanted to be involved in their design. Further details on each notion, including examples from the transcripts, can be found in Appendix A (Tables S2 and S3).

### 4.1 The four notions

The four notions are organized below in order of relevant stage in the life cycle of an orphan drug. Refer to Appendix A to see the thematic networks behind each notion (Figures S2 ‐S5) and for further details on the life cycle (Figure S6).

#### 4.1.1 All stakeholders have roles and responsibilities in the orphan drug life cycle

Patients and caregivers believed that all stakeholders *“[have] a role and… a responsibility” (Patient 5, Workshop 1 or P5, W1)* within the orphan drug life cycle *(“I'm sorry, it's like if you don't go to vote, you can't complain!” – Caregiver 1, W1 or C1, W1)*, and saw themselves as experts in the “lived” experience. At the same time, they described challenges in this role, which related to the physician–patient relationship. Patients and caregivers often face pushback from physicians who are *“not open… [And] don't always listen” (P5, W2)* to information patients/caregivers have gathered through their own research and experiences.

In general, patient organizations were viewed as trusted representatives of and important information conduits for the disease community. As such, they are well positioned to identify and inform patients about opportunities to be involved in the life cycle (eg to provide input into coverage decision making). When asked whether there is a role for patient organizations in educating and managing expectations of patients and caregivers around new therapies for which there may be limited clinical evidence, patients and caregivers responded *“absolutely.”* They also felt that those who formally or informally contribute to decision making within the life cycle should share their knowledge and insights with the rest of the disease community.

With respect to the role of physicians, patients and caregivers felt that physicians should be responsible for ensuring their patients are aware of all treatment options, regardless of the cost. Several expressed frustrations with physicians who *“didn't read the literature” (P2, W2)* and struggled to effectively provide care due to unfamiliarity with the rare disease.

#### 4.1.2 Research on rare diseases and orphan drugs is challenging

Patients and caregivers reiterated the challenges involved in conducting research on rare diseases and orphan drugs, the most significant of which remains the poorly understood natural histories of rare diseases (“*Well finally, at least we know I'm not the only one…” – P5, W2*) and its impact on the discovery of effective therapies. They emphasized the importance of on‐going collection of natural history and clinical outcomes data. They recognized that while registries may play a role, they require significant resources to implement and maintain. *“Many drugs can't support that type of registry”* and there are *“the physicians as well…they don't have time to fill out the paper work” (P3, W2)* for on‐going data collection.

Patients and caregivers also identified challenges involved in conducting clinical trials on orphan drugs in Canada. In their view, trials are *“not likely to be happening in Canada” (P4, W2)*, which is a concern, as they represent an important means of obtaining early access to new therapies. Regardless of location, trials are limited by small patient sample sizes (*“we didn't have 99 patients that were going to enroll”— C2, W2)* and a lack of validated outcome measures.

#### 4.1.3 Challenges around coverage decision‐making processes affect access to orphan drugs

Patients and caregivers discussed challenges in Canadian coverage decision‐making processes that affect their ability to access orphan drugs. They appreciated that coverage decision making is complicated by the significant uncertainties that exist around the clinical benefit of orphan drugs when one is *“not quite sure how it's going to be used and what the outcomes will be…” (P1, W2)*. However, they questioned why such processes do not routinely involve specialist physicians *“who know something about the disease and the drug” (C1, W2)*. They also acknowledged the high cost of orphan drugs as an added challenge for decision‐makers, as well as desperate demands from patients and families for access to treatments with *“no data to support [them] whatsoever…” (P4, W1)*. These demands are often exacerbated by “*inequality [in access] across Canada” (P7, W1)*, which they blamed on the lack of a national health‐care system. They believed that provincial control of health‐care budgets has hindered the implementation of nation‐wide programmes, like the pan‐Canadian Pharmaceutical Alliance (pCPA; established to conduct joint drug plan negotiations for brand drugs in Canada), which could directly affect access to orphan drugs.

There is also a lack of transparency in drug coverage decision making. One participant wondered: “*Why don't they have accountability? Why is there no transparency there?” (P6, W1*). Patients and caregivers felt that they were purposefully kept in the dark by those involved who *“don't want [patients/caregivers] to know” (P1, W1)*.

Finally, patients and families discussed how Canada represents only a small share of the global drug market and is “*not a friendly place for [pharmaceutical companies] to come to” (P8, W1)*. As a result, there is a need for the government to introduce policy mechanisms for bringing new drugs into Canada that provide some security for companies *“around how long they have to recoup that money” (P8, W1)*.

#### 4.1.4 All patients are unique

Patients and caregivers explained that no single patient can represent the views of the entire disease group because all patients are “*unique”* and do *“not [have] just one experience” (P1, W1) “You can't do… one size fits all [with orphan drugs]” (P5, W1)*. Further, rare diseases are often heterogeneous, with symptoms, severity and response to treatment varying across patients who share a diagnosis (*“…we're not having the same bodies. We don't have the same ways [of] metabolizing [drugs]” (P3, W2))*. *“You're dealing with all ages, you're dealing with different responses to treatment, different lifestyle… at least in our area I would feel very bad as a patient representative to be the only one saying what I think are the right outcomes” (P1, W2)*.

### 4.2 Aspects of an ideal MAP

Additionally, patients' and caregivers' described the components that a MAP should contain. Six aspects of an ideal MAP were identified. In considering how to operationalize these components, a checklist was developed, which organized the aspects into three categories relating to accountability, governance and evidence collection (Table 1). An annotated version of this checklist can be found in Appendix B, which maps the notions onto the checklist components (Table B1).

**Table 1:** Checklist for the characteristics of an Ideal MAP

Accountability		
Programme Goals		
Is the MAP appropriate for the question at hand?	□ Yes	□ No
Will the patients receive earlier access to the drug?	□ Yes	□ No
Will the programme collect the evidence needed to find the right drug for the right patient?	□ Yes	□ No
Will all of the processes within the programme (eg decision making) be transparent to ensure greater buy‐in?	□ Yes	□ No
Governance		
Programme‐Specific Committee		
Will there be a programme‐specific committee established to guide the MAP?	□ Yes	□ No
Will there be 3 patient members on the committee?	□ Yes	□ No
Will the patient members meet the follow criteria:		
Meet a minimum level of experience with the health‐care system	□ Yes	□ No
Have a meaningful role on the committee?	□ Yes	□ No
Are accountable back to the disease community that they represent?	□ Yes	□ No
Will patient organizations select the patient members?	□ Yes	□ No
Will there be a physician committee member?	□ Yes	□ No
Will they be an expert in the rare disease?	□ Yes	□ No
Will patient organizations select the physician member?	□ Yes	□ No
Will the committee meetings be open to all patients and caregivers who wish to attend?	□ Yes	□ No
Individual Patient Input		
Will individual input from a broad range of patients be collected to develop the MAP?	□ Yes	□ No
Will the process be quick and efficient?	□ Yes	□ No
Will there be a variety of ways for patients to provide input?	□ Yes	□ No
Will the input processes be transparent and patients well informed of the opportunity?	□ Yes	□ No
International Collaboration		
Will there be collaboration with other countries to learn from their experiences with MAPs?	□ Yes	□ No
Will there be collaboration with other countries to conduct trials (if necessary)?	□ Yes	□ No
Will there be collaboration with experts in other countries to educate Canadian physicians on the rare disease?	□ Yes	□ No
Evidence Collection		
On‐going Monitoring and Registries		
Will there be on‐going monitoring with an engaged physician and good documentation (eg through EMRs)?	□ Yes	□ No
Will the following information be collected:	□ Yes	□ No
Natural history data?	□ Yes	□ No
Qualitative data?	□ Yes	□ No
Clinical outcomes?	□ Yes	□ No
Outcome Measures and Continuation Criteria		
Will the outcome measures used be meaningful to patients and adequately capture their experiences?	□ Yes	□ No
Will patients provide input on meaningful outcome measures?	□ Yes	□ No
Will decisions to continue/discontinue therapy be made between physicians and patients without the use of set continuation criteria?	□ Yes	□ No
Will there be follow‐through on the results of the MAP?	□ Yes	□ No

#### 4.2.1 Accountability aspects

##### Programme goals

While patients and caregivers viewed MAPs as enabling earlier access to potentially effective therapies, they wanted to ensure that this option is used appropriately or *“for the right purpose” (C1, W2)*—it must be able to address research questions aimed at determining the right dose of the right drug for the right patient. Individualized treatment protocols, which involve *“trying [the drug] on each individual patient and seeing if it's working for them or not” (C3, W2),* may be required.

Transparency in all aspects of the MAP was emphasized. This includes opportunities for patient and caregiver input; as many felt that other stakeholders do not effectively communicate, their requests for input and so the patients *“don't know when these [opportunities] happen.”* It was felt that transparent MAPs would improve patients' acceptance of treatment decisions by helping them *“under[stand] the process a little more and [take] the sting out of why [they] can't get medication” (P4, W1)*.

#### 4.2.2 Governance aspects

##### MAP‐specific Committee

Patients and caregivers indicated that MAPs should be overseen by a MAP‐specific committee with *“a stipulation that there's patient representation” (P9, W1)* from three patient members who: 1) meet a minimum level of experience within the health‐care system, 2) have a meaningful role on the committee and 3) are accountable back to the organization they represent to avoid bias and enhance knowledge translation. To this end, they saw a role for patient organizations in selecting patient representatives who *“understand all [their] needs… [to] go on [their] behalf” (P3, W1)*.

They also agreed that committees should include a physician who specializes in the specific rare disease—*”somebody in the medical field who understands [the specific disease]” (P4, W1)* and the patient community should select that physician. Finally, there was a widely held view that committee meetings should be “*open to anybody” (P7, W1)* so that all patients/caregivers have the opportunity to provide input into the programme. This is discussed in detail below.

##### Individual patient input

The importance of providing opportunities for *individual* patient and caregiver input in the development of a MAP was stressed *(“the patient, in whatever format, deserves a voice” (P2, W1))*. Further, such input must be collected through a process that is quick, efficient and accessible *(“thinking about the people who are at home with their disease and can't get to meetings, but want to have a voice, if they have a computer and a family member they can sit beside them and put their answers in it”—P3, W1)*. Several approaches to collecting feedback were discussed, including online surveys, written documents, videos or face‐to‐face interviews with the committee. While most agreed that online surveys are an effective and efficient way of gathering input from as many patients as possible, some felt there should still be an opportunity for an individual to present to a committee (ie *“the choice of doing it in person*”—P6, W1).

##### International collaboration

Given the nature of rare diseases, patients and caregivers proposed collaborating with other countries on certain MAPs, using the experiences of patients across multiple countries. They provided the example of patients who have participated in clinical trials based out of the United States and wondered if for MAPs there could be *“a parallel sort of thing where the [Canadian] physician enters [data] into… [a] US database” (P4, W2)*. Also, there may be opportunities to learn from other countries that use MAPs and potentially *“adopt one of [their] systems” (P1, W1)*.

#### 4.2.3 Evidence collection aspects

##### Outcome measures and continuation criteria

Patients and caregivers believed they should have an opportunity to provide input on the outcome measures selected and used as continuation criteria to ensure they are meaningful. They felt that patients should be asked, “*What do you think? What else can you tell us?” (C1, W2*). With respect to continuation criteria, where these could not be determined *a priori* (eg for poorly understood, ultra‐rare, heterogeneous diseases), participants felt that decisions around continuation on therapy should be made through a conversation between patients and their physicians (eg *“try and up the dose”—P4, W2*).

At the same time, the need to *“act on the answer” (P1, W2)* provided through a MAP was stressed by patients and caregivers. Where the treatment proves ineffective based on previously agreed outcomes, participants indicated it should be discontinued, with decision‐makers enforcing follow‐through.

“..so you do have to set schemes up in such a way (1) in hope of getting an answer and (2) that you're going to act on the results in a reasonable kind of way.” (C2, W2)

##### On‐going monitoring and registries

Patients and caregivers felt that MAPs must have “*a documentation process in place” (P2, W2)* to support on‐going monitoring with an engaged physician and data collection should begin before treatment is started (eg natural history registries) to identify “*the stages of that patient journey… [and] the progressions” (C1, W2)* of the disease. Once treatment has begun, registries should collect qualitative and quantitative data related to the impact of the drug.

#### 4.2.4 Disease/drug priorities

Patients and caregivers recognized that health‐care resources are finite and that it is infeasible to have a MAP for every drug. As such, they also considered possible disease or drug eligibility criteria for deciding when to use a MAP. They included drugs that treat *“life‐threatening or chronically debilitating conditions” (P2, W1)* and those for which there are no other legitimate alternatives (ie when an alternative exists but is not an option for all patients, eg, due to intolerance). Drugs that are innovative (ie offer a new mechanism of action) or high cost were also seen as priorities. When asked whether disease prevalence alone is a sufficient criterion to make a drug a priority for a MAP, patients and caregivers both responded “no.” While there was broad agreement from patients and caregivers on these criteria, some wondered “*why we…are even thinking about excluding [drugs]” (P4, W1)*, arguing that the use of MAPs for all drugs may make the health‐care system more efficient.

### 4.3 The role of MAPs

In addition to the 4 notions described above, 3 overarching “sentiments” emerged. These captured why patients and caregivers felt MAPs were a reasonable solution for addressing the uncertainties that coverage decision‐makers face. While the workshops were not intended to gather information on why patients and caregivers supported the use of MAPs, these sentiments were embedded throughout their discussions around what MAPs should look like.

First, trust in other stakeholders is often lacking. Patients and caregivers questioned the meaningfulness of many outcome measures used in decision‐making processes that they feel are opaque (*“Maybe they don't want us to know” – P4, W1)*. Some did not trust their physicians to know which therapies were most appropriate. One patient described receiving a prescription that she later *“found out… [they] should have never… had” (P7, W2)*. Also, at times, they felt that their physician chose not to inform them of all their treatment options because of the costs. “*Physicians [make] treatment decisions based on the cost of the drug” (P2, W1),* not on its potential effectiveness, assuming *“[the patient] can't afford it.”*

The second “sentiment” was desperation. Patients and families described feeling desperate to find a treatment for their disease, particularly when no alternatives exist. They become willing to try almost anything and accept risk thresholds that are much higher than those accepted by their health‐care providers. They *“[are] emotional, [and they] want to get better” (P1, W1)* so if they are offered access to a drug in a trial or a MAP *“[they'll] sign anything”* (*P9, W1)* to participate.

The third “sentiment” that emerged was hope. Patients and caregivers were steadfast in their belief that access to orphan drugs can be improved. One caregiver said: “*I'm not giving up for anything. And if my son doesn't make it, I'll also be fighting for the other ones” (C2, W1)*. This theme was apparent in both patients' and caregivers' enthusiasm around the workshop dedicated to the design of an ideal MAP and in their discussions about uncertainty and the difficulty it creates for decision‐makers. They recognized that these uncertainties are an issue, but felt there are ways they could be involved to help reduce them (“*Let's get [it] done…”—C2, W1)*, such as identifying meaningful outcome measures (*“…it's fairly easy to ask the [patients] what they would see as success”—P1, W2)* and contributing to decision‐making committees.

## 5 DISCUSSION

The work described in this study contributes to the growing body of literature supporting the inclusion of patients in the assessment of new health technologies to inform reimbursement decisions. To date, much of the attention has been focused on the development of generic guidance documents for patient involvement in HTA, such as those developed by the European Patients Academy (EUPATI).^17^, ^18^ Several of the items on the MAP checklist are consistent with these documents. For example, the EUPATI calls for the nomination of patient and clinical experts by patient organizations to serve on HTA committees. They are also consistent with The European Organization for Rare Diseases' Charter that provides principles for collaboration between sponsors and patient organizations.^19^

The MAPs checklist is an example of a tool co‐designed by patients and caregivers to not only improve access to high‐cost drugs for rare diseases, but also generate the kind of evidence needed to inform appropriate reimbursement (ie right drug for the right patient at the right time). To our knowledge, no other such tool exists. However, some jurisdictions, such as England and Wales, have already implemented MAP‐like schemes [ie patient access schemes (PAS)]. While PAS proposals from pharmaceutical companies are not co‐designed by patients or caregivers, their input is sought during the review process managed by the National Institute for Health and Clinical Excellence (NICE).^10^ Those that take the form of a “complex scheme,” where pricing and reimbursement is outcome‐based, share many of the elements presented in the MAPs checklist.

Additionally, a checklist for evaluating access with evidence development (AED) schemes, which serve a similar purpose to MAPs, has been published.^20^ Specifically, AEDs provide interim coverage to patients through participation in a study designed to generate evidence needed to make a definitive coverage decision. The elements are broadly similar to those of the MAPs checklist.

While much of this work focussed on what an ideal MAP should look like, general discussions around the current context of orphan drug access, the challenges that patients and caregivers face and, ultimately, why MAPs was viewed as an appropriate solution also took place. The three sentiments identified from these discussions (trust, hope and desperation) have been documented in published literature. One study found that patients with lower levels of trust in their physician were more likely to want an autonomous role in treatment decision making.^21^ Another study which involved a qualitative analysis of cancer patients' conversations demonstrated that hope often served as a justification for action.^22^ Finally, a recent ethics paper argued that it is a combination of desperation and hope that motivates patients with untreatable diseases to drastic measures to find potentially effective therapies.^23^

It was also recognized that MAPs will not address all of the issues that patients and families face with respect to managing rare diseases (eg the exclusion of patients and specialists with relevant expertise from committees reviewing submissions for drug coverage or disparities in access to coverage across Canadian provinces and territories). Studies on the reasonableness of patients and their willingness to accept limits have been documented in other studies.^24^, ^25^

### 5.1 Limitations

Both workshops were held at national events hosted by CORD, and it is possible that the individuals who chose to participate in these events were not representative of the rare disease population in Canada. However, CORD is comprised of over 80 different rare disease patient organizations and covers travel expenses for patients and caregivers to attend their events, reducing the likelihood of bias.

## 6 CONCLUSION

The MAP checklist co‐designed by patients and caregivers offers a tool for informing the development and evaluation of such policy options, which aim to improve access to drugs where there is a high degree of uncertainty in the available evidence. Future research is needed to examine the feasibility of this checklist and roles for other stakeholders.

## CONFLICTS OF INTEREST

A. Young, D. Menon, J. Street, W. Al‐Hertani and T. Stafinski report no conflict of interests.
