# Supplementary material for: A checklist for managed access programmes for reimbursement co‐designed by Canadian patients and caregivers
Source: Health Expect. 2018 Apr 6;21(6):973–80. doi: 10.1111/hex.12690 (PMC6250858; doi:10.1111/hex.12690)
Supplement: Supplementary file 1 [file HEX-21-973-s001.docx]

**Appendix A**

| **Figure S1. Progression of research conducted in collaboration with CORD.** | | | |
| --- | --- | --- | --- |
|  | **Multi-stakeholder deliberative discussions** |  |  |
|  |  | *General overview of ways in which patients and their families should be involved in an ideal access framework for orphan drugs* | |
|  | **Patient and caregiver Deliberative discussion** |  |  |
|  |  | *In-depth review of ways in which patients and their families want to be involved in the orphan drug lifecycle to help reduce the uncertainties that decision-makers face* | |
|  | **Patient and caregiver webinar** |  |  |
|  |  | *Validation of findings from the stakeholder workshops and deliberative discussion* | |
|  | **Ways in which Canadian patients and their families want to be involved in the orphan drug lifecycle** |  |  |
|  |  | *Patient and caregiver priorities for involvement identified with emphasis on MAPs* | |
|  | **Patient and caregiver workshops** |  |  |
|  |  | *Exploring what a tool for the development of MAPs might look like when designed by patients and caregivers* | |
|  | **Checklist for MAPs** |  |  |
|  |  |  |  |

**Figure S2. Thematic Network 1: all stakeholders have roles and responsibilities.**

**Figure S3. Thematic Network 2: all patients are unique.**

**Figure S4. Thematic Network 3: there are weaknesses in the existing healthcare system.**

**Figure S5. Thematic Network 4: research on rare diseases and orphan drugs is challenging.**

| **Figure S6. Lifecycle of a drug.** | | | | | | |
| --- | --- | --- | --- | --- | --- | --- |
| **Pre-clinical phase** | **Clinical trials** | **Regulatory approval** | **Real-world studies** | **Reimbursement decision-making** | **Routine clinical use** | **Replacement with new therapies** |
| **All stakeholders have roles and responsibilities in the orphan drug lifecycle** | **Research on rare diseases and orphan drugs is challenging** |  |  | **Healthcare system weaknesses affect the orphan drug lifecycle** |  |  |
|  |  |  |  | **All patients are unique** |  |  |

| **Table S1. Questions presented during workshops.** | |
| --- | --- |
| 1. **When should we use MAPs?** | 1. Is there anything about the disease that makes MAPs a funding option? 2. Is there anything about the drug that you think makes it a good candidate for a MAP? |
| 1. **What should a MAP look like?** | 1. Who should be involved in determining the conditions of the MAP (i.e., patient eligibility criteria)? 2. Who decides on what data should be collected? 3. What would that process look like? 4. What do you think the role of the patient advocacy group is for a MAP? 5. What would you expect as part of a MAP? 6. How do we arrive at stopping criteria for a MAP? 7. How do we make sure that everybody will abide by the conditions of the MAP? |

| **Table S2. Basic, organizing, and global themes identified in the thematic network analysis.** | | | | |
| --- | --- | --- | --- | --- |
| **FG** | **Codes** | **Basic Themes** | **Quotes** | **Organizing Themes** |
| ***All stakeholders have roles and responsibilities within the orphan drug lifecycle*** | | | | |
| 1 | Patients and families know the patients’ experiences best | Patients and their families are disease experts | *“I’m living with that body. I know me.”* | Unique expertise |
| 2 | Patients and families know which outcomes matter most |  |  |  |
| 2 | Patient organizations know the patients | Patient organizations are most familiar with the disease community and the patients involved in it | *“…And that’s what I tell patients to ask the physicians to do, to call, for example, [patient organization] where we see hundreds of these patients as one physician in Quebec may see…”*  *“They may have already had the experience and know what to do.”* |  |
| 1, 2 | Patient autonomy in decision-making | Patients are responsible for deciding how to approach their care and which treatments to take | *“And it’s totally the patients who will take themselves off the drugs because it is not worth it whatsoever.”*  *“…and I make corrections to the medication…”* | Expectations of stakeholders |
| 1, 2 | Patients are responsible for managing their own care |  |  |  |
| 1 | Patients are responsible for getting involved | Patients are responsible for getting more involved throughout the orphan drug lifecycle (e.g. advocacy; decision-making processes, etc.) | *“All the people at home are saying I don’t have to because somebody is going to do it for me, somebody’s going to do it for me, somebody’s going to do it for me. And then, they’ve lost their right. I’m sorry, it’s like if you don’t go to vote, you can’t complain!”* |  |
| 1 | Involved patients communicate with their disease community | Patients who become more involved have a role in sharing their knowledge with the rest of the disease community | *“And that’s where I think it’s important that the rest of us are there for those voices so that we can go back to the community, can go back to these people and say…”*  *“I’m going to give you this information and what you do with it is up to you, kind of thing.”*  *“In your case you’ve got this great organization now. You can bring it back to there and then it’s up to these people to take that information and do whatever they want with it.”* |  |
| 1 | Family members and/or caregivers should help patients with their treatment decision-making when necessary (e.g. walking them through decision-making tools) | Family members and/or caregivers should help patients with their treatment decision-making when necessary (e.g. walking them through decision-making tools) | *“I can see that coming in handy like if you take a look at how long you need and I can only speak from my own situation, I’m the only person who knows my son. I’m the only one who knows all of his medications. Everything from head to toe, inside and outside. God forbid something should ever happen to me, there would be this that his sister could go “I recognize this, I didn’t know that.”* |  |
| 1 | Patient organizations are responsible for identifying patients and informing them of opportunities to be involved | Patient organizations are responsible for identifying and informing patients about opportunities to be involved (e.g. in decision-making processes) | *“Through the support groups.”*  *“Could you do it through patient support groups?”* |  |
| 1 | Patient organizations are responsible for managing patients’ expectations | Patient organizations are responsible for managing patients’ expectations of new orphan drugs | ***“Do you think there’s a role for patient organizations in helping patients to figure out what would be reasonable to push for or not?”***  *“Absolutely.”* |  |
| 1 | Patient organizations are responsible for educating their patients | Patient organizations are responsible for educating patients and physicians through decision-making tools and educational documents (e.g. infographics) | *“So, yeah I do believe that there’s a responsibility of the patient group to educate.”* |  |
| 1 | Patient organizations educate through decision-making tools |  |  |  |
| 1 | Improving patient care through education/decision-making tools |  |  |  |
| 1 | Patient organizations should educate physicians |  |  |  |
| 1 | Patient organizations educate through educational documents |  |  |  |
| 1 | Physicians are responsible for informing patients of opportunities to be involved | Physicians are responsible for informing patients about all opportunities to be involved (e.g. in decision-making processes) | *“Through the physicians, through the clinics, through support groups, through foundations.”* |  |
| 1 | Physicians are responsible for informing patients about all available drugs | Physicians are responsible for informing their patients about all drugs that are available, regardless of their price | *“We’re developing it because we want our physicians to know, because we know so many of our physicians are making treatment decisions based on cost of the drug.”*  *“They won’t prescribe this medication…‘I won’t even mention it to them because they can’t afford it’.”* |  |
| 1 | Lack of support due to unfamiliarity with rare diseases (e.g. in charity runs) | Rare diseases receive less support because they are unknown to the general population | *“But every disease needs support.”* | Barriers faced in fulfilling roles and responsibilities |
| 1 | Patients face different barriers to involvement (mental, physical, emotional) | Patient involvement in the lifecycle is limited by a number of different barriers (e.g. physical, mental, emotional) | *“If you’re not capable, if you’re not cognitively able to do it…”*  *“But on the other side of it, like when I said that the majority of our patients can’t help they need help. And that is true.”*  *“The majority need help. And so I don’t want anyone…like where do you start judging where we are accountable and that…”*  *“But at the same time, at the end of the day, the patient is a patient. So when you have 2 or 3, you still have to expect sometimes one of these are not going to be feeling well. And you know, like it’s…that’s part of their lives.”* |  |
| 2 | Physicians are not open to patients’ expertise | Physicians are not open to patients’ expertise | *“They’re not open.”* |  |
| 1 | Some family members lack knowledge about the disease and treatments | Not all familiar members are familiar with rare diseases and the treatment protocols | *“…but nobody knows what she’s dying of or what’s really going on.”*  *“Like what she’s taking or what she’s…anything.”* |  |
| 1 | Patient organizations have a limited capacity to advocate properly | Patient organizations have a limited capacity to advocate properly and often struggle to maintain member support | *“…I’d always get the ones where somebody just died, somebody just got diagnosed…there out there that first year running for Aunt May and ‘I’m gonna do this’ and then after their mourning process is done, they’re gone!”* |  |
| 1 | Patient organizations taking on too much |  |  |  |
| 1 | Patient organizations struggle to maintain member support |  |  |  |
| 2 | General practitioners are not always familiar with rare diseases and their treatments | General practitioners are not always familiar with rare diseases and their treatments | *“…and what happened was they spent hours in meetings where the [general practitioners] and the other physicians were questioning the basic principles and measures and indicators of this 60 year old disease. So it was a very bad experience for these medical geneticists that treat at the [disease] clinic and that know…”*  *“He didn’t read the literature. Next going into hypoglycemic shock and nobody’s picking up on it.”*  *“So some physicians could be very uncomfortable with dealing with these one-on-one personalized cases with treatments.”* |  |
| 2 | Some physicians are unfamiliar with rare diseases |  |  |  |
| ***Research on rare diseases and orphan drugs is challenging*** | | | | |
| 2 | Lack of validated outcome measures | There are a lack of validated outcome measures for rare diseases | *“What would be doable because if we propose full blown managed access schemes but all would need patient registries and have validated quality of life indicators and patient input and all that…it’s not going to happen.”* | Orphan drug research |
| 2 | Small sample sizes | Clinical trials on orphan drugs are often limited by small patient sample sizes | *“If we don’t have that, if we don’t have enough patients to do that, if we can’t validate that, does that mean then that we aren’t going to get anything?”*  *“Finally they came back and said we’d have to have 99 patients. Well we didn’t have 99 patients that we’re going to enroll.”* |  |
| 2 | Clinical trials are less likely to take place in Canada | Clinical trials do not frequently take place in Canada for orphan drugs | *“Thinking of that from an ultra-rare disease perspective where the clinical trials are not likely to be happening in Canada…”* |  |
| 2 | Registries are often infeasible | Registries are often infeasible | *“What would be doable because if we propose full blown managed access schemes but all would need patient registries and have validated quality of life indicators and patient input and all that…it’s not going to happen.”* |  |
| 2 | The natural histories of rare diseases are often poorly understood | The natural histories of rare diseases are often poorly understood | *“And now, 10 years later we know other patients having that.”*  *“I was afraid, I was scared of like everything.”* | Rare disease research |
| 2 | Registries are often infeasible | Registries are often infeasible | *“What would be doable because if we propose full blown managed access schemes but all would need patient registries and have validated quality of life indicators and patient input and all that…it’s not going to happen”*  *“However, if it’s a complex… the way we have it now, many drugs can’t support to have that type of registry that you’re asking [for]. It’s often on the manufacturer so…and the physician as well. It’s long, it’s complex, you know, they don’t have time to fill out that paper work.”* |  |
| ***Challenges around coverage decision-making processes affect access to orphan drugs*** | | | | |
| 1 | Drug pricing is done through secret negotiations | Drug pricing is done through secret negotiations | *“It’s true. Secret negotiations with the drug companies…”*  *“Why don’t they have accountability? Why is there no transparency there? I don’t get that.”*  *“We never know what the deal is.”* | Lack of transparency |
| 1 | Patients and family members are unaware of the system, how decisions are made and opportunities for involvement within it | Patients, families, and physicians are unfamiliar with the decision-making processes in the orphan drug lifecycle | *“I didn’t even…I’m a nurse and I didn’t even know about this.”*  *“I’ve learned so much in these two days about these processes but like these three people who would represent whatever…”*  *“So they didn’t know what the system is.”*  *“They don’t. My doctor, she couldn’t tell me *unintelligible* all I know that it’s going to be way more expensive than growth hormone.”*  *“And they simply don’t know…so…”* |  |
| 1 | Physicians are unfamiliar with the system and how decisions are made |  |  |  |
| 1 | Belief that other stakeholders want patients in the dark on decision-making processes | Belief that other stakeholders want patients in the dark on decision-making processes | *“Maybe they don’t want us to know.”*  *“No, they don’t! They don’t want us to know.”* |  |
| 1 | Good follow-up through EMRs | If EMRs could be successfully implemented they would be helpful in providing continuity of care and good data collection | *“Could you imagine the access to records?”*  *“You can go into anyone of the hospitals and they can pull up your file, your last week in endocrinology, they can pull up your neurosurgery…”*  *“Everything? Could you imagine one system? Fantastic!”*  *“You know, done a debate and come up with one and it was mandatory for everybody. Could you imagine the data collection?”* | Challenges in implementing EMRs |
| 1 | Continuity of care through EMRs |  |  |  |
| 1 | Unified EMR system |  |  |  |
| 1 | Good data collection through EMRs |  |  |  |
| 1 | Resistance to EMRs from physicians | Challenges of ongoing monitoring include issues around EMRs (e.g. resistance from physicians; ownership disagreements) and the feasibility of establishing registries | ***“…a lot of the push back came from clinicians. They perceived it as other people more easily checking on…”***  *“Big brothers watching you.”*  *“God forbid you’re accountable.”* |  |
| 1 | Access to/ownership of EMRs |  | *“I’ve never signed a document to say that the doctor is the only person that can keep my records.”* |  |
| 2 | Feasibility of registries is an issue |  | *“What would be doable because if we propose full blown managed access schemes but all would need patient registries and have validated quality of life indicators and patient input and all that…it’s not going to happen.”* |  |
| 1 | Public outcry, particularly through social media, demanding access to drugs that are not necessarily effective | Public outcry is used to obtain access to orphan drugs that are not necessarily effective | *“…no data to support this whatsoever and yet it was, you know, huge public outcry about it.”* | Challenges in accessing orphan drugs |
| 2 | Orphan drugs are usually more expensive | Orphan drugs are often expensive | *“…the drugs that are coming in as orphan in our experience are all very expensive drugs...”* |  |
| 2 | Greater uncertainty around clinical benefit | There is greater uncertainty around the clinical benefit of orphan drugs | *“So you’re not quite sure how it’s going to be used and what the outcomes will be depending on the way it’s used.”* |  |
| 2 | Existing coverage review and decision-making processes do not necessarily involve true disease experts | Existing coverage review and decision-making processes do not necessarily involve true disease experts | *“…a big enhancement is that we’re actually going to bring in some people who know something about the disease and the drug and ask them to comment on this… like really?”* |  |
| 1 | Provincial inequalities in drug access | Provincial inequalities in drug access | *“So this is where I come in and say we’ve got inequality across Canada.”*  *“It’s awful. It has to be one.”* | No national healthcare system |
| 1 | Provinces have their own budget | Provinces have their own budget | *“The problem is the provinces have all their own money.”* |  |
| 1 |  | Small Canadian drug market | *“Because we need these companies to bring the drugs to Canada. Like why do you think we only have one treatment right now or one special access? Canada is not a friendly place for them to come to. If they don’t have some security around how long they have to recoup that money, why would they come to Canada?”* | Small Canadian drug market |
| ***All patients are unique*** | | | | |
| 1, 2 | Patients respond differently to treatments | Patients with the same disease respond differently to treatments | *“…we’re not having the same bodies. We don’t have the same ways [of] metabolizing.”*  *“A one size decision does not fit all in many of these cases…”* | Patients have very different experiences with their diseases |
| 2 | Rare diseases are heterogeneous | Rare diseases are highly heterogeneous | *“Everybody’s different.”*  *“…and I learn so much from everybody else too, because we’re not just one experience. Everyone is unique.”* |  |
| 2 | Rare diseases vary in severity |  |  |  |
| 2 | Rare diseases manifest differently in different patients |  |  |  |
| 1 | Patients at different stages of progressive diseases |  |  |  |
| 1 | Inappropriate stopping criteria | Outcome measures used in clinical trials or for stopping criteria in real-world studies often do not capture the benefits that patients experience | *“You can’t do a one size fits all.”* | Patients have different values |
| 2 | Clinical outcome measures do not capture treatment benefits that patients experience |  |  |  |
| 2 | Patients have different ideas of what is a meaningful benefit | Patients have different interpretations of what is a meaningful benefit | *“You’re dealing with all ages, you’re dealing with different response to treatment, different lifestyle…at least in our area I would feel very bad as a patient representative to be the only one saying what I think are the right outcomes.”* |  |
| ***Best practice – what does an ideal managed access program look like?*** | | | | |
| 1 | Right drug for the right patient | Identify the right drug for the right patient | *“… does a patient need all 15 of these medications?… It’s in my blister pack, I don’t know. Right? And it’s like…you probably don’t actually need it.”*  *“So for me, and again an obvious fact, that we should be trying it on each individual patient and seeing if it’s working or not for them.”*  *“So that patient should at least try it. So that’s what I mean also. It’s not just the validity of the test and the other ones who walk less should also be trying it also in case, you know…”*  *“Because a one size decision does not fit all in many of these cases…”* | Goals |
| 2 | Appropriate dose |  |  |  |
| 2 | All patients should try |  |  |  |
| 2 | Let all patients try to identify those for whom it will work |  |  |  |
| 2 | Individualized treatment |  |  |  |
| 1 | Avoiding disease progression due to slow access | Early access | *“The failure is that if it causes the patient to have to trial this medication and fail and within that time they lost their kidneys where you could have started them on this drug and they could have saved their kidneys…we don’t want that either, right?”* |  |
| 2 | Right scheme for the right purpose | Program appropriateness | *“Quite frankly, it goes back to what you’re saying here, you gotta choose the right scheme though for the right purpose.”*  *“The scheme has got to be designed for the purpose.”*  *“There’s no hope in hell of getting that. So you set that basis of the study in a way, you’re never…and then they abandoned the study after 3 years, 6 years…whatever you want to call it.”*  *“Nobody is willing to go there so you do have to set schemes up in such a way (1) in hope of getting an answer and (2) that you’re going to act on the results in a reasonable kind of way.”*  *“I like your question…not a stupid question but a smart question…”* |  |
| 2 | Program appropriateness |  |  |  |
| 2 | Program appropriateness for question at hand |  |  |  |
| 1 | Transparent program | A transparent program that improves the acceptance of decisions by educating patients on the decision-making process | *“It really took the sting out of why I can’t get my medication because I understood the process a little more.”*  *“That was a huge lesson I’m going home with. Has it changed my mind? No, but do I have a much better understanding? Absolutely.”* |  |
| 1 | Improving acceptance of decisions through education on decision-making process |  |  |  |
| 1, 2 | All drugs/diseases to improve system efficiency | While putting all drugs through a MAP may improve system efficiency, prioritizing certain drugs will make the introduction of MAPs more manageable and avoid an overwhelming burden/cost of too many MAPs | *“So if they save a lot of money there, they have a lot more money available for rare disease.”*  *“I don’t really see why we have these exclusions or are even thinking about excluding stuff.”*  ***“I think just to make it more manageable. To say okay let’s try and bite off this piece first.”*** | Drug/disease priorities |
| 1 | Prioritize certain drugs to make MAPs more manageable |  |  |  |
| 1 | Prioritize certain drugs because of the associated burden/cost of MAPs |  |  |  |
| 1 | Life-threatening or chronically debilitating | Drugs that treat life-threatening or chronically debilitating diseases | *“It also has to be a life threatening or chronically debilitating condition.”* |  |
| 1 | Not just prevalence | Criteria beyond prevalence | ***“But then there’s others, like the Netherlands or Belgium, where it’s not just prevalence.”*** |  |
| 1, 2 | No alternatives | No legitimate alternatives | *“There might be a disease modifying kind of thing but what if it’s really either very expensive also…”*  *“…or really hard to adhere to or there’s other problems with it.”*  *“…because technically there’s something out there but in real life…if you could use it…”*  ***“Okay, so something around those two criteria and figuring out a way to talk about an alternative that is actually a legitimate alternative.”***  *“That’s intolerance. I think we need to say failure or intolerance.”*  ***“…you can’t say that supportive care is the equivalent of having an option.”*** |  |
| 1 | No affordable alternative |  |  |  |
| 1 | No manageable alternative |  |  |  |
| 1 | Supportive care is not an alternative |  |  |  |
| 1 | Failure on alternatives |  |  |  |
| 1 | Intolerance to alternatives |  |  |  |
| 1 | No legitimate alternative |  |  |  |
| 2 | New mechanism of action | Innovative drugs | *“Well my first one would be when the drug is somewhat innovative and it works in a different way, either in terms of how it’s prescribed.”*  *“Well in the case that I’m thinking about, it’s not a different mechanism but it’s a different half-life.”* |  |
| 2 | Different half life |  |  |  |
| 2 | Expensive orphan drugs | Expensive orphan drugs | *“And that was my question because not all orphan drugs are necessarily expensive so, you know, that’s a factor when you think of the burden…it shouldn’t be all drugs.”*  *“If it’s a really cheap therapy and it’s going to cost more to do an access scheme than it is to basically, you know, give the drug then follow the drug…yeah, why bother?”* |  |
| 2 | Drugs with greater uncertainties in clinical benefit | Drugs with greater uncertainty in clinical benefit | *“…if it’s straightforward, we know what the outcomes are going to be, we know what patients should be on it or at least reasonably…if it’s a really cheap therapy and it’s going to cost more to do an access scheme than it is to basically, you know, give the drug then follow the drug…yeah, why bother?”* |  |
| 1 | Patient input on drugs/diseases for MAPs | Patient input is essential to help identify appropriate diseases/drugs for MAPs | *“Proves that patient input is so important.”* |  |
| 1 | Patient input to ensure MAP “makes sense” | MAPs guided by a program-specific committee with 3 patient members who meet a minimum level of experience with the healthcare system, have a meaningful role on the committee, and are accountable back to the community they represent to avoid bias | ***“And then there would have to be for each MAP, or each managed access plan, we’d have to have a way of getting the really, the important, detailed feedback on what actually makes sense or not.”*** | Program-specific committee |
| 1 | Patient membership on program-specific committee |  | *“So when you have these committees that fall together, there needs to be a stipulation that there’s patient representative…representation on that board.”*  ***“And actually now that I’m thinking about it, do you actually need an overriding group above that? Because I don’t actually think you do.”*** |  |
| 1, 2 | Need for broad patient representation on committees |  | *“And then you may need within that, maybe you do want someone who has had a transplant, may you do want someone who has…you know, I think you’ve gotta be…you can’t do a one size fits all.”* |  |
| 1 | Multiple patient representatives on committee with set term |  | *“What I would suggest is that you would have more than one patient and of course they would have like a term.”* |  |
| 1 | “Real” patients on the committee |  | *“But when I mention that by saying the consumer, we actually have to specify that it’s a patient. Someone who has life dependency on this medication.”*  *“That’s what I was trying to say, you have to like an informed patient. Someone who can actually talk on behalf or at least understand the situation.”*  *“And then you also want to be very sure that you have a kind of rotating person who is knowledgeable in the particular drug area or disease area that’s under discussion.”*  *“They just have to be well educated. Self-educated.”*  *“It’s gotta be somebody who is a chronic user of the system so they completely understand how it works.”*  *“There have to be criteria and that criteria has to be followed for the qualifications of the people who are on it.”*  *“I don’t think there will be a one size fits all, that’s why I am saying you have to look at what is under discussion and make sure that ask yourself what are the important criteria that we want to make sure they know.”*  *“I personally believe you always want somebody who uses the system all the time.”* |  |
| 1 | Patient committee member needs to have a certain level of experience with the healthcare system |  |  |  |
| 1 | “Informed” patient committee member |  |  |  |
| 1 | Patient member is chronic system user |  |  |  |
| 1 | 3 patient committee members |  | *“Well the first thing is you can never send one patient. All the research shows, actually believe it or not, the best is 3 patients. Not even 2, but 3. So I think we should start by asking for best practice, and we have that but it’s to show that that’s best practice.”* |  |
| 1 | Patient committee member responsible back to community to avoid bias |  | *“You have to have at least one of those patients that’s responsible back to the community in some way…* *so they’ve gotta be on a board of an organization, have been elected in a sense by the community to represent them”*  *“…and I prefer all three, has some accountability back to their community. So if we’re not happy, you know, if you get in a situation where one person is way off in la-la-land because some drug company sent them on a trip somewhere, they are accountable back to their community. The community can say excuse me, out you go. I think you need that too.”* |  |
| 1 | Avoiding biased patient committee members |  | *“That’s where my concern is and sometimes what I worry about is pharma influence. We want their support, yes we want their support, we want their help, we do want to work with them… but we want to make sure that we have an objective opinion of patients not somebody that’s been influenced.”* |  |
| 1 | Meaningful role for committee members to avoid tokenism |  | *“It’s totally tokenism.”*  *“It’s a pat on the head.”*  *“The patient reps would sit there and say nothing and it was absolute crap.”* |  |
| 1 | Patient committee member selected by patient organizations | Patient committee members selected by patient organizations | *“We all agree that [patient name] go for us and speak on…like do we all, do you think she understands all our needs and all our… everything and she can go on our behalf?”* |  |
| 1 | Committee meetings open for all patients to attend | Open committee meetings, which all patients and families are permitted to attend | *“I think it should always be open to anybody if they wanted to attend.”*  *“If I wanted to take the time, to have the option to get there, I would like that option.”* |  |
| 1 | Physician committee member | Physician committee member on program-specific committee | *“Do you also have your physician accompany as another representative?”*  *“Well somebody who’s in the medical field who understands…”*  *“…and I think it was you who first mentioned it in Toronto, is that it’s also important…it’s also important that patients help to identify their experts because someone had mentioned to me that in a lot of the cases for the CADTH reviews, there really is no true clinical expert representing the actual… who understands disease and on that committee…”* |  |
| 2 | Physician committee member who is a disease specialist |  |  |  |
| 1 | Patient input to ensure MAP “makes sense” | Individual patient input from a broad range of patients to help develop an appropriate MAP | ***“And then there would have to be for each MAP, or each managed access plan, we’d have to have a way of getting the really, the important, detailed feedback on what actually makes sense or not.”***  *“A temporary portal or whatever, so that this is where you go in and fill out these answers and then that information is directly going to go into, you know, that part. Whether it goes to the patient advocate and they take it, those three patient advocates, or whether it goes straight into the session.”*  *“I think that there should be a patient group that represents but I think that the patient, in whatever format, deserves a voice.”*  *“…you can get some more individual input.”*  *“I think it’s really important that the patient is part of all those things.”*  *“And so do you have to have…you don’t have to have a separate process but you do have to I think… is you gotta have enough of a representation of patients that you can get that range of inputs.”* | Individual patient input |
| 1 | Individual patient input |  |  |  |
| 1, 2 | Need for broad individual patient input |  |  |  |
| 1 | Quick & efficient patient input | Input collected through a quick and efficient process | ***“Like, do you think that if they opened up some kind of web portal that you could type that in so that it would be an easy way for government to have that information when they’re trying to set up…because you don’t want it to be so burdensome that they take bloody well three months to get the patient input that they need in order to start this. You want it to be quick, right?”*** |  |
| 1 | Opportunity for all patients to provide input through written document or video | Accessible ways for patients to provide individual input | *“You could also do it electronically.”*  *“… to have the choice of doing it in person”*  *“I’d want a face-to-face. To really communicate.”*  *“Thinking about the people who are maybe at home with their disease and can’t get to meetings, but want to have a voice and if they have a computer and a family member, they could sit beside it and put their answers in.”*  *“Like gather up all the information and it’s the one voice?”* |  |
| 1, 2 | Opportunity to provide input through online survey |  |  |  |
| 1 | Opportunity to provide input face-to-face |  |  |  |
| 1 | Accessible ways for patients to provide input |  |  |  |
| 1 | Collect broad individual patient input through patient organizations |  |  |  |
| 1 | Transparent input process | Transparent | *“It’s gotta come through transparency. Thinking about the people who are maybe at home with their disease and can’t get to meetings, but want to have a voice and if they have a computer and a family member, they could sit beside it and put their answers in. We have this technology right, so that’s an example of using it for good.”*  *“So, also…so when this is happening again it comes with transparency, because we don’t know when these things happen, right?”* |  |
| 2 | Collaboration with other countries to conduct trials | Collaboration with other countries to conduct trials | *“…or that you do a parallel sort of thing where the physician enters into say the US database.”*  *“Thinking of that from an ultra-rare disease perspective where the clinical trials are not likely to be happening in Canada, is it possible to have something, there are trials in the States going on that we know about, so that maybe we could get some Canadian patients matched with a Canadian physician that could be part of the trial in the States.”*  *“Working together internationally.”* | International collaboration |
| 1 | Learning from other countries about MAPs | Learning from other countries about MAPs | *“Have there been any other pilot programs in other countries to find out what was in their managed access systems and which countries has it worked and why can’t we follow or adopt one of those systems?”* |  |
| 1 | Collaborating with other countries to educate Canadian physicians on rare diseases | Collaborating with other countries to educate Canadian physicians on rare diseases | *“And so that you get learning in Canada for the physician in a rare disease or ultra-rare disease…”* |  |
| 2 | Ongoing monitoring with engaged physician | MAPs require ongoing monitoring with an engaged physician and good documentation | *“…where I’ve had to take myself off the medication and go to a clinic and say, what do I do because the reactions are so bad but then the moment that happened when I call his office and say “can I get an earlier appointment, this is what happened” they try and fit me. If they can’t…they still.”*  *“Part of managed access is to have a documentation process in place for when you’re doing these things so that it’s captured.”* | Ongoing monitoring and registries |
| 2 | Ongoing monitoring with good documentation |  |  |  |
| 2 | Collection of qualitative data | Data collection should begin before treatment (i.e. natural history data) and include the qualitative data in addition to clinical outcomes | *“You can actually get some qualitative information at the different stages of the patient journey.”*  *“Map out for each one… that disease, what are the stages of that patient journey, what are the progressions…”*  *“Send it out there widely and ask people to respond to it and grow that database.”* |  |
| 2 | Natural history registries |  |  |  |
| 1 | Good follow-up through EMRs | Using EMRs to consistently follow-up on patients, providing continuity of care and good data collection | *“Could you imagine the access to records?”*  *“You can go into anyone of the hospitals and they can pull up your file, your last week in endocrinology, they can pull up your neurosurgery…”*  *“Everything? Could you imagine one system? Fantastic!”*  *“You know, done a debate and come up with one and it was mandatory for everybody. Could you imagine the data collection?”* |  |
| 1 | Continuity of care through EMRs |  |  |  |
| 1 | Unified EMR system |  |  |  |
| 1 | Good data collection through EMRs |  |  |  |
| 2 | Collecting meaningful outcome measures | Collect meaningful outcome measures that capture the patients’ experiences | *“…ask us what outcomes we’re looking for is the first thing and, you know, fairly easy as long as you know who the patients are, it’s fairly easy to ask them what they would see as success.”*  *“Here’s what we’re thinking in terms of outcomes, what do you think?”* | Outcome measures and continuation criteria |
| 2 | Outcomes that capture patients’ experiences |  |  |  |
| 2 | Patient input on meaningful outcome measures | Patient input on meaningful outcome measures and continuation criteria | *“Here’s what we’re thinking in terms of outcomes, what do you think? What else can you tell us? Because quite frankly if you don’t do that, you’re going to build this program and at the end of the day everybody is going to say ‘well, that kind of sucks, we don’t wanna but into that.’”*  *“That’s where patient input is very important on…so for…reconsidering.”*  *“Because a one size decision does not fit all in many of these cases…”*  *“Ideally the patients will come in at the clinical trial stage, I mean, and that clinical trial input would actually lead over into regulatory approvals.”* |  |
| 2 | Patient input on continuation criteria |  |  |  |
| 2 | Early involvement in the lifecycle to get input on outcome measures |  |  |  |
| 2 | Harms are clear stopping criteria | Harms are clear stopping criteria | *“Yeah, if you get an adverse effect, if you get a harm. Yeah, those may be worth stopping for them.”* |  |
| 2 | Consider other factors (e.g. inappropriate dosing) before stopping | Instead of stopping criteria, decisions to continue on with a therapy should be made through a conversation between physicians and patients | *“The question might be do I need to up the dosage? Do I need to make it more frequent? Do I need to do more in order to get the impact?”*  *“I can continue with observation for another period of time. Or I can up the dosage. Or I can add something else. You know, I think we need to be clear, what are the options there?”*  *“…like we always talk about, okay this is what it’s supposed to do…if it doesn’t work after this amount of time, we’ll try and up the dose but we can only go up to this amount of medications and after this point… and we have to try and move on to something else…and these are the types of side-effects and if the side-effects are too much, then I go right back say ‘listen, this is too great.’”* |  |
| 2 | Decision points (i.e. a conversation between physician and patient) instead of set stopping criteria |  |  |  |
| 2 | Follow-through on results of MAP | Follow-through on the results of the MAP | *“Nobody is willing to go there so you do have to set schemes up in such a way (1) in hope of getting an answer and (2) that you’re going to act on the results in a reasonable kind of way.”*  *“A smart question and you act on the answer.”* |  |
| Note: bolded quotes are statements made by the facilitator which the patients and caregivers verbally agreed with. | | | | |

| **Table S3. Notions behind a MAP mapped onto the types of uncertainty.** | | | | | | | |
| --- | --- | --- | --- | --- | --- | --- | --- |
| **Statements and sub-themes** | | **Uncertainties** | | | | | |
|  |  | **Clinical benefit** | **Value for money** | **Adoption/ diffusion** | **Affordability** | **Availability** | **Access** |
| **All stakeholders have roles and responsibilities within the orphan drug lifecycle** | |  |  |  |  |  |  |
| *Expectations of stakeholders* | |  |  |  |  |  |  |
|  | Patients are responsible for deciding how to approach their care |  |  |  |  |  |  |
|  |  |  |  |  |  |  |  |
|  | Patients are responsible for getting more involved in the lifecycle |  |  |  |  |  |  |
|  |  |  |  |  |  |  |  |
|  | Patients who become more involved must share their knowledge with their disease community |  |  |  |  |  |  |
|  |  |  |  |  |  |  |  |
|  | Family members and/or caregivers should help patients with treatment decisions when necessary |  |  |  |  |  |  |
|  |  |  |  |  |  |  |  |
|  | Patient organizations are responsible for identifying and informing patients about opportunities to be involved |  |  |  |  |  |  |
|  |  |  |  |  |  |  |  |
|  | Patient organizations are responsible for managing patients' expectations |  |  |  |  |  |  |
|  |  |  |  |  |  |  |  |
|  | Patient organizations are responsible for educating patient and physicians through decision-making tools and educational materials |  |  |  |  |  |  |
|  |  |  |  |  |  |  |  |
|  | Physicians must inform patients about all available treatments, regardless of cost |  |  |  |  |  |  |
|  |  |  |  |  |  |  |  |
|  | Physicians must inform patient about all opportunities to be involved in the lifecycle |  |  |  |  |  |  |
|  |  |  |  |  |  |  |  |
| *Unique stakeholder expertise* | |  |  |  |  |  |  |
|  | Patients and their caregivers are disease experts |  |  |  |  |  |  |
|  |  |  |  |  |  |  |  |
|  | Patient organizations know the disease community & patients within it the best |  |  |  |  |  |  |
|  |  |  |  |  |  |  |  |
| *Challenges face in fulfilling roles and responsibilities* | |  |  |  |  |  |  |
|  | Patients face physical, mental, and emotional barriers to involvement |  |  |  |  |  |  |
|  |  |  |  |  |  |  |  |
|  | Physicians do not respect patients’ expertise |  |  |  |  |  |  |
|  |  |  |  |  |  |  |  |
|  | Patient organizations have a limited capacity to advocate effectively and struggle to maintain member support |  |  |  |  |  |  |
|  |  |  |  |  |  |  |  |
|  | Rare disease communities receive less support because they are lesser known to the general population |  |  |  |  |  |  |
|  |  |  |  |  |  |  |  |
|  | General practitioners are not always familiar with rare diseases and their treatments |  |  |  |  |  |  |
|  |  |  |  |  |  |  |  |
|  | Family members are not always familiar with the disease and treatment protocols |  |  |  |  |  |  |
|  |  |  |  |  |  |  |  |
| **All patients are unique** | |  |  |  |  |  |  |
| *Patients experience their diseases very differently* | |  |  |  |  |  |  |
|  | Patients with the same disease respond differently to the same treatment |  |  |  |  |  |  |
|  |  |  |  |  |  |  |  |
|  | Rare diseases are highly heterogeneous in how they manifest |  |  |  |  |  |  |
| *Patients have different values than each other and other stakeholders* | |  |  |  |  |  |  |
|  | Outcome measures and stopping criteria often do not capture the benefits that patients feel they experience |  |  |  |  |  |  |
|  |  |  |  |  |  |  |  |
|  | Patients have different interpretations of meaningful benefits |  |  |  |  |  |  |
| **There are weaknesses in the existing healthcare system** | |  |  |  |  |  |  |
| *Lack of transparency* | |  |  |  |  |  |  |
|  | Drug pricing negotiations are done in secret |  |  |  |  |  |  |
|  |  |  |  |  |  |  |  |
|  | Patients, caregivers, and physicians are now well-informed on decision-making processes |  |  |  |  |  |  |
|  |  |  |  |  |  |  |  |
|  | Patients and caregivers do not trust decision-makers to be transparent |  |  |  |  |  |  |
| *Access to orphan drugs* | |  |  |  |  |  |  |
|  | Public outcry is used to obtain access to drugs that are not necessarily effective |  |  |  |  |  |  |
|  |  |  |  |  |  |  |  |
|  | Orphan drugs are expensive |  |  |  |  |  |  |
|  |  |  |  |  |  |  |  |
|  | Greater uncertainty around clinical benefit |  |  |  |  |  |  |
|  |  |  |  |  |  |  |  |
|  | Coverage decision-making does not always involve disease experts |  |  |  |  |  |  |
| *No national healthcare system* | |  |  |  |  |  |  |
|  | Inequality in drug access across the provinces |  |  |  |  |  |  |
|  |  |  |  |  |  |  |  |
|  | Provinces control their own budget |  |  |  |  |  |  |
| *Issues with use of electronic medical records* | |  |  |  |  |  |  |
|  | No unified EMR system |  |  |  |  |  |  |
|  |  |  |  |  |  |  |  |
|  | Resistance from physicians to EMR use |  |  |  |  |  |  |
|  |  |  |  |  |  |  |  |
|  | Patient frustration over EMR ownership |  |  |  |  |  |  |
| *Canada's small drug market is less attractive to pharmaceutical companies* | |  |  |  |  |  |  |
| **Research on rare diseases and orphan drugs is challenging** | |  |  |  |  |  |  |
| *Research on orphan drugs* | |  |  |  |  |  |  |
|  | Trials are limited by small sample sizes |  |  |  |  |  |  |
|  |  |  |  |  |  |  |  |
|  | Treatment registries are often infeasible |  |  |  |  |  |  |
|  |  |  |  |  |  |  |  |
|  | Lack of validated outcome measures |  |  |  |  |  |  |
|  |  |  |  |  |  |  |  |
|  | Fewer clinical trials in Canada |  |  |  |  |  |  |
| *Research on rare diseases* | |  |  |  |  |  |  |
|  | Natural histories of rare diseases are often poorly understood |  |  |  |  |  |  |
|  |  |  |  |  |  |  |  |
|  | Natural history registries are often infeasible |  |  |  |  |  |  |
