# Supplementary material for: A checklist for managed access programmes for reimbursement co‐designed by Canadian patients and caregivers
Source: Health Expect. 2018 Apr 6;21(6):973–80. doi: 10.1111/hex.12690 (PMC6250858; doi:10.1111/hex.12690)
Supplement: Supplementary file 2 [file HEX-21-973-s002.docx]

| **Table B1. Notions behind a MAP mapped onto the characteristics of an ideal MAP.** | | | | |
| --- | --- | --- | --- | --- |
| **Characteristics** | | | | **Notions*** |
| ***Accountability*** | | | |  |
| **Program Goals** | | | |  |
| • Is the MAP appropriate for the question at hand? | | 🞎 Yes | 🞎 No | 3^a^ |
| • Will the patients receive earlier access to the drug? | | 🞎 Yes | 🞎 No | 3^b^ |
| • Will the program collect the evidence needed to find the right drug for the right patient? | | 🞎 Yes | 🞎 No | 4^c^ |
| • Will all of the processes within the program (e.g. decision-making) be transparent to ensure greater buy-in? | | 🞎 Yes | 🞎 No | 3^d^ |
| ***Governance*** | | | |  |
| **MAP-Specific Committee** | | | |  |
| • Will there be a MAP-specific committee established to guide the MAP? | | 🞎 Yes | 🞎 No | 1, 3^e^ |
| • Will there be 3 patient members on the committee? | | 🞎 Yes | 🞎 No | 1, 4^f^ |
| • Will the patient members meet the follow criteria: | |  |  |  |
|  | • Meet a minimum level of experience with the healthcare system | 🞎 Yes | 🞎 No | 1^g^ |
|  | • Have a meaningful role on the committee? | 🞎 Yes | 🞎 No | 1^h^ |
|  | • Are accountable back to the disease community that they represent? | 🞎 Yes | 🞎 No | 1,4^i^ |
| • Will patient organizations select the patient members? | | 🞎 Yes | 🞎 No | 1, 4^j^ |
| • Will there be a physician committee member? | | 🞎 Yes | 🞎 No | 1^k^ |
| • Will they be an expert in the rare disease? | | 🞎 Yes | 🞎 No | 1, 3, 4^l^ |
| • Will patient organizations select the physician member? | | 🞎 Yes | 🞎 No | 1, 3 ,4^m^ |
| • Will the committee meetings be open to all patients and caregivers who wish to attend? | | 🞎 Yes | 🞎 No | 1, 3^n^ |
| **Individual Patient Input** | | | |  |
| • Will individual input from a broad range of patients be collected to develop the MAP? | | 🞎 Yes | 🞎 No | 1, 4^o^ |
| • Will the process be quick and efficient? | | 🞎 Yes | 🞎 No | 3,4^p^ |
| • Will there be a variety of ways for patients to provide input? | | 🞎 Yes | 🞎 No | 4^q^ |
| • Will the input processes be transparent and patients well informed of the opportunity? | | 🞎 Yes | 🞎 No | 3^r^ |
| **International Collaboration** | | | |  |
| • Will there be collaboration with other countries to learn from their experiences with MAPs? | | 🞎 Yes | 🞎 No | 3^s^ |
| • Will there be collaboration with other countries to conduct trials (if necessary)? | | 🞎 Yes | 🞎 No | 2, 3^t^ |
| • Will there be collaboration with experts in other countries to educate Canadian physicians on the rare disease? | | 🞎 Yes | 🞎 No | 1, 4^u^ |
| ***Evidence Collection*** | | | |  |
| **Ongoing Monitoring and Registries** | | | |  |
| • Will there be ongoing monitoring with an engaged physician and good documentation (e.g. through EMRs)? | | 🞎 Yes | 🞎 No | 1, 2^v^ |
| • Will the following information be collected: | | 🞎 Yes | 🞎 No |  |
|  | • Natural history data? | 🞎 Yes | 🞎 No | 2^w^ |
|  | • Qualitative data? | 🞎 Yes | 🞎 No | 2^x^ |
|  | • Clinical outcomes? | 🞎 Yes | 🞎 No | 2^y^ |
| **Outcome Measures and Continuation Criteria** | | | |  |
| • Will the outcome measures used be meaningful to patients and adequately capture their experiences? | | 🞎 Yes | 🞎 No | 2, 4^z^ |
| • Will patients provide input on meaningful outcome measures? | | 🞎 Yes | 🞎 No | 1, 2, 4^aa^ |
| • Will decisions to continue/discontinue therapy be made between physicians and patients without the use of set continuation criteria? | | 🞎 Yes | 🞎 No | 1, 4^bb^ |
| • Will the findings of the MAP impact decision-making? | | 🞎 Yes | 🞎 No | 3^cc^ |
| *This column outlines the notions underpinning each aspect of the checklist. The notions are as follows: 1) all stakeholders have roles and responsibilities; 2) research on rare diseases and orphan drugs is challenging; 3) challenges around coverage decision-making processes affect access to orphan drugs; 4) all patients are unique. See below for further details. | | | | |

a. Patients and caregivers acknowledged the challenges involved in coverage decision-making and recognized that not all of these challenges can be addressed through MAPs.

b. Patients and caregivers viewed MAPs as a mechanism for improving access, which they identified as one of the main challenges related to coverage decision-making.

c. Patients and caregivers indicated that MAPs need to be flexible enough to ensure that they generate the kind of evidence needed to inform decisions that take into account disease heterogeneity across patient populations.

d. Patients and caregivers felt that there is a lack of transparency in existing coverage decision-making processes.

e. Individuals who represent different stakeholder groups on committees tasked with developing a MAP need to have relevant expertise. Specifically, patients and caregivers mentioned the important role they play in providing input, since they are experts in the ‘lived’ experience, and that no single individual would be able to represent the views of all rare disease patients.

f. The appointment of multiple patient members was viewed as way of addressing concerns around disease heterogeneity.

g. Patients are experts in living with their disease, which for many involves substantial and ongoing interactions with the healthcare system. A patient who is newly diagnosed or has an early or mild form of a disease may not have developed that experience.

h. Patients believed that they have roles and responsibilities in coverage decision-making, but also reflected on their experiences on decision-making committees when they felt their roles were tokenistic. To avoid tokenism in committees developing MAPs, decision-makers and other stakeholder committee members must respect the role of the patient members and the expertise they have to offer.

i. Patient committee members are responsible for representing not only themselves, but also a community of patients with unique experiences. As such, patients and caregivers specified that the patient committee members must be accountable back to the community to ensure the community feels accurately represented and can take action if not. For example, patient members could have their positions revoked if the community feels misrepresented.

j. Patients and caregivers viewed patient organizations as trusted advocates who are well-positioned to identify individuals to serve on MAP-specific committees.

k. Patients and caregivers indicated that all stakeholders, including physicians, have roles and responsibilities in coverage decision-making. Therefore, physicians need to be a part of MAP committees.

l. Patients and caregivers also expressed the importance of involving physicians with expertise in the management of their diseases, which often involve multiple organ systems, complex symptoms, and unclear natural histories.

m. Patients and caregivers expressed a lack of trust in coverage decision-making processes. To improve trust, they proposed that patient organizations nominate a disease specialist trusted by the patient community to serve as the physician committee member.

n. Patients and caregivers indicated that one way of dealing with the lack of transparency in coverage decision-making would be to allow them the opportunity to attend committee meetings as observers.

o. Patients and caregivers indicated that, because of disease heterogeneity, symptoms, severity, and response to treatment often vary. Thus, there is a need for a process that seeks input from a broad range of patients and caregivers.

p. Patients and caregivers expressed concerns over the timeliness of coverage decision-making. Therefore, they wanted to ensure that any mechanisms for incorporating patient input would not lengthen the process.

q. Patients and caregivers discussed the fact that mechanisms for eliciting their input need to be multimodal. For example, some patients may not be able to provide written input, while others may only be able to provide written input. Some may feel most comfortable sharing their story, while others may prefer to address a specific set of questions.

r. Patients and caregivers stressed that transparency includes assurance that they will be informed of opportunities for input.

s. Patients and caregivers referred to presentations on international initiatives for improving access to therapies and felt that Canadian decision-makers should embrace opportunities to learn from those initiatives.

t. Patients and caregivers were aware of clinical trials in other countries and proposed that decision-makers in Canada consider collaborating with those countries in order to generate the evidence needed to reduce decision uncertainties. For example, arrangements that enable patients in Canada to join those trials through a shared funding arrangement were suggested.

u. Patients and caregivers mentioned the role that international centres of excellence in certain rare diseases could play in cases where expertise in Canada is lacking.

v. Patients and caregivers stressed the importance of improving data collection on rare diseases and the impact of therapies through MAPs. They indicated that this requires not only willing patients and caregivers, but also engaged physicians who ensure ongoing monitoring and documentation of clinical outcomes and/or disease progression.

w. Patients and caregivers viewed MAPs as a mechanism for increasing knowledge related to the natural history of a rare disease.

x. Patients and caregivers felt that trials often focus on quantitative clinical measures that do not reflect their preferences. They proposed that MAPs incorporate qualitative or subjective measures that better capture what matters to them.

y. Patients and caregivers argued that MAPs enable the collection of data that aren’t traditionally a part of clinical trials.

z. Patients and caregivers expressed concerns over the potential selection of a rigid set of outcome measures that fails to account for disease heterogeneity.

aa. Patients and caregivers mentioned that much of the research on rare diseases does not include outcomes that are meaningful to them. In their view, this could be addressed through their involvement in the selection of outcome measures during the development of research protocol.

bb. Patients and families reiterated the need to ensure flexibility in the establishment of continuation criteria. Given the heterogeneity of rare diseases and that many patients and families often experience the same disease differently, they should be involved in decisions to continue/discontinue therapy.

cc. Patients and caregivers saw MAPs as a way to address the challenges that currently impede decision-making, allowing for earlier access to promising therapies and greater certainty in coverage decisions.
